# Caregiver Stigma and Burden in Memory Disorders: An Evaluation of the Effects of Caregiver Type and Gender

**DOI:** 10.1155/2016/8316045

**Published:** 2016-01-28

**Authors:** Phoebe V. Kahn, Heather A. Wishart, Jennifer S. Randolph, Robert B. Santulli

**Affiliations:** ^1^Geisel School of Medicine at Dartmouth, Hanover, NH 03755, USA; ^2^Dartmouth-Hitchcock Medical Center, Lebanon, NH 03756-0001, USA

## Abstract

Despite considerable gains in public awareness of dementia, dementia patients and their caregivers continue to be stigmatized. Previous work has explored stigma and burden among adult children of persons with dementia in Israel, but no similar data exist for spousal caregivers or caregivers in general in the United States. This study examines the differences in stigma and burden experienced by spousal and adult child caregivers and male and female caregivers of persons with dementia. Eighty-two caregivers were given the Zarit Burden Inventory Short Form (ZBI) and the Caregiver Section of the Family Stigma in Alzheimer's Disease Scale (FS-ADS-C). Scores on the FS-ADS-C and ZBI were positively correlated (*r*_*s*_ = .51, *p* < .001). Female caregivers reported experiencing more stigma on the FS-ADS-C (*t*(80) = −4.37, *p* < .001) and more burden on the ZBI (*t*(80) = −2.68, *p* = .009) compared to male caregivers, and adult child caregivers reported experiencing more stigma on the FS-ADS-C (*t*(30.8) = −2.22, *p* = .034) and more burden on the ZBI (*t*(80) = −2.65, *p* = .010) than spousal caregivers. These results reinforce the importance of support for caregivers, particularly adult child and female caregivers who may experience higher levels of stigma and burden.

## 1. Introduction

Alzheimer's disease and other dementias are a growing public health crisis. There are currently 5.3 million people who suffer from Alzheimer's disease. If no cure or prevention for Alzheimer's disease is found, the prevalence is estimated to more than triple by 2050 [1]. Alzheimer's profoundly affects not only individuals with the disease but also the caregivers who dedicate copious time, energy, and emotion to caring for their loved ones with Alzheimer's. It can be a devastating illness for caregivers who bear its emotional, physical, and financial burdens. In 2014, nearly 16 million caregivers provided 18 billion hours of unpaid care to those with Alzheimer's and other dementias [1]. Importantly, eighty-five percent of caregivers for the elderly in the United States are family members [2].

A number of studies have examined factors that influence caregiver burden and the repercussions of caregiver burden (e.g., [3, 4]). Severity of disease, frequency and intensity of care, and lack of help are associated with increased burden [3]. However, few studies have examined stigma as a factor impacting caregiver burden. Stigma has been defined as an “attribute, behavior or reputation which is socially discrediting in a particular way: it causes an individual to be mentally classified by others in an undesirable, rejected stereotype rather than in an accepted, normal one” [5].

In many cases, family members become victims of stigma and may experience feelings of shame about the disease as well. The feeling of stigma experienced by patients and caregivers is an important and potentially modifiable contributor to caregiver burden [6]. Unfortunately, research in general and quantitative studies in particular in the area of dementia and stigma are limited. Alzheimer's Disease International conducted an online survey in which they asked caregivers “Have you been avoided or treated differently?” Caregivers most commonly answered that they experienced social exclusion [7].

A few qualitative studies have investigated the effect of stigma on dementia caregivers in Asian families. One study found that, among Vietnamese and Chinese dementia caregivers, 81% of which were daughters and daughters in law, stigma was strongly associated with negative stereotypes of the elderly and characteristics of chronic illness [8]. Another study showed that caregiver stigma among Chinese caregivers of persons with severe dementia influenced caregiver functioning and wellness. In addition, a study in Israel showed that family stigma may prevent adult child, spousal, and other family caregivers from seeking services for their loved ones [9]. Werner et al. [10] also conducted a qualitative study in which they examined family stigma experienced by 10 adult children caregivers of persons with Alzheimer's disease.

Based on the findings of this work, as well as previous literature, Werner and colleagues developed the Family Stigma in Alzheimer's Disease Scale (FS-ADS), a quantitative, structured questionnaire about family stigma. The FS-ADS contains 62 items designed to assess three dimensions of family stigma: caregivers' stigma, lay persons' stigma, and structural stigma. Initial findings in a population of 185 adult child caregivers of noninstitutionalized persons indicated that the FS-ADS is reliable and valid in assessing Alzheimer's disease family stigma [11].

More recently, Werner et al. [6] explored the relationship between family stigma and caregiver burden among adult children of persons with Alzheimer's disease in Israel, making use of the FS-ADS. This study was the first quantitative study examining this relationship. Caregiver stigma was defined as the dimension of family stigma that includes the cognitive attributions, emotional reactions, and behavioral responses of caregivers to their loved ones with Alzheimer's. The results suggested that caregiver stigma increased caregiver burden for those caring for family members with Alzheimer's disease more than either layperson or structural stigma. An important recommendation of this study was that psychosocial interventions should target stigmatic beliefs to reduce caregiver burden. There has been no research, however, that further refines which caregivers might benefit most from these interventions, and no similar data exist for spousal caregivers or caregivers in general in the United States.

This study expands on prior work by examining the relationship of caregiver stigma and caregiver burden in a sample of both adult child and spousal caregivers in the United States. Additionally, the study compares the degree of caregiver stigma and burden experienced by caregivers based on their type (spousal or adult child) and gender, to enhance our understanding of which groups of caregivers may be most susceptible.

## 2. Methods

### 2.1. Participants

The study sample included 82 adult child or spousal caregivers recruited from the Dartmouth Memory Clinic and Dartmouth-sponsored caregiver support groups who were primary caregivers of a family member with a dementia or memory disorder diagnosis. Of the participants, 59 were spousal caregivers and 23 were adult child caregivers. Patients were characterized as having a diagnosis of dementia in 90.1% of cases, with about half of those specifically reported as Alzheimer's disease. The remaining 9.8% were noted to have significant memory disorders, but an official diagnosis of dementia was not recorded (diagnosis information was missing for one participant). The characteristics of the participants are shown in Table 1.

### 2.2. Measures

All caregivers were given the caregiver stigma section of the FS-ADS [11], the Zarit Burden Interview Short Form (ZBI; [12]), and were asked basic demographic and other questions.

Caregiver stigma was assessed using a modified version of the caregiver section of the FS-ADS (FS-ADS-C). The entire 18-item caregiver section of the scale was administered to participants in its original format, with respondents being asked to rate each item using a Likert-type scale of 1 to 5, reflecting the extent to which they felt the item applied to them (with 1 being the lowest and 5 being the highest). Higher scores are reflective of greater stigma. However, given that the scale was administered to both adult child and spousal caregivers, there were wording issues that necessitated four questions about concealment to be dropped prior to analyses. For example, one question inquiring about concealment of the patient from siblings identified different relatives of the patient depending on whether the participant was an adult child or a spousal caregiver. Furthermore, since many caregivers in this study did not live with the patient, two items relating to the provision of assistance with ADL and IADL were also dropped. Cronbach's alpha for the final, 12-item modified version of the FS-ADS-C was .69.

The Zarit Burden Interview (ZBI) Short Form was used to assess caregiver burden. The full version of the ZBI (22 items) was pared down in 2001 to create Short (12 items) and Screening (4 items) versions, resulting in interviews that were easier to administer yet retained results similar to that of the full version [12]. The interview questions are rated on a 5-point Likert scale of 0–4, with 0 being “rarely” and 4 being “nearly always,” with higher scores indicating greater caregiver burden. Cronbach's alpha for the Short, 12-item version used in our study was .87.

In addition to the two questionnaires described above, caregiver participants were asked a number of demographic and other questions, including age, sex, relationship to the person with dementia, whether the participant lives with the person with dementia, and years of education. Questions about the person with dementia included diagnosis, time since diagnosis, and time since symptoms were first noticed. Finally, participants were asked whether or not they felt that they or their family member with dementia had been stigmatized because of their dementia and, if so, what they felt would be helpful in diminishing stigma.

### 2.3. Procedures

After consenting to participate in the study, in accordance with procedures approved by the Committee for the Protection of Human Subjects of Dartmouth College, participants were given the opportunity to complete the questionnaires by phone or by mail. If the questionnaires were completed by phone, all questions were answered during one telephone session. If the questionnaires were completed by mail, the participants were provided preaddressed, stamped envelopes in which they returned the completed questionnaires.

Fifty-one caregivers were interviewed over the telephone, and an additional 31 caregivers completed questionnaires that were returned by mail. Twenty spousal caregivers responded by mail (33.9% of total spouses) and 39 by phone (66.1% of total spouses), whereas 11 adult children responded by mail (47.8% of total children) and 12 by phone (52.2% of total children). There were no significant differences in the proportions of phone and mail responders by caregiver type or gender, as assessed by Pearson's chi-square tests (all *p* > .05; data not shown).

## 3. Statistical Analysis

Spearman's rank order correlation analyses were used to assess the relationship between caregiver stigma (FS-ADS-C score) and caregiver burden (ZBI score). Group differences were assessed using independent *t*-tests, Pearson's chi-square tests, and Fisher exact tests, as appropriate. All statistical analyses were conducted using IBM SPSS Statistics 23.

## 4. Results

As shown in Table 1, adult child and spousal caregivers in the sample differed significantly on some characteristics. As expected, the caregivers differed on age, with spousal caregivers being 20 years older, on average, than adult child caregivers. Spousal caregivers were more likely to live with the patient for whom they provided care, while adult child caregivers were more likely to be female. No significant differences were noted on years of caregiver education, diagnosis of patient, time since patient symptom onset, or time since patient diagnosis.

Across the entire sample, scores on the FS-ADS-C and ZBI were positively correlated (*r*_*s*_ = .51, *p* < .001), indicating that, as caregiver stigma increases, caregiver burden increases. This correlation remained significant within caregiver type and gender groups (all *p* < .05; data not shown).

Means and standard deviations for the FS-ADS-C and ZBI scores are presented in Table 2 for the entire study sample, as well as by caregiver type and caregiver gender. In comparing caregivers by gender, female caregivers reported experiencing more stigma on the FS-ADS-C (*t*(80) = −4.37, *p* < .001) and more burden on the ZBI (*t*(80) = −2.68, *p* = .009) compared to male caregivers. Comparing type of caregiver, adult children caregivers experienced significantly greater stigma on the FS-ADS-C (*t*(30.8) = −2.22, *p* = .034) and burden on the ZBI (*t*(80) = −2.65, *p* = .010) compared to spousal caregivers.

Notably, there was a disproportionately low number of males among the adult child caregiver sample (13.0% male) as compared to spousal caregivers (35.6% male) (*χ* ^2^(1, *N* = 82) = 4.07, *p* = .044), making it unclear as to whether observed caregiver type differences may in fact be due to the disproportionate number of females in the adult child group.

Seventy-seven (94%) caregivers responded to a question regarding what they felt would be helpful in diminishing stigma associated with dementia. Of these, 32 (42%) stressed education of the general public as well as the health care community, and 22 (29%) emphasized the importance of contact and communication with those who have the disease.

## 5. Discussion

The number of persons with memory disorders is dramatically rising. With this increase, many more millions of caregivers' lives will also change because of this disease. It is crucial to recognize the difficult tasks these caregivers face and identify interventions to ameliorate their burden that has been well described in numerous studies [3, 4, 6]. Stigma has arisen in the literature as one potential target. Our study further supports this view.

Although several studies have qualitatively examined caregiver stigma (e.g., [8, 13]) and one study in Israel [6] has quantified the effects of family stigma on caregiver burden, this study is the first to assess both caregiver type and gender in relation to stigma and burden. It is also the first study in the United States to quantify effects of caregiver stigma on caregiver burden.

The current study found that caregiver stigma and caregiver burden were correlated, consistent with Werner's findings in Israel. It was found that female caregivers experienced more caregiver burden and stigma than male caregivers. While adult child caregivers did show higher scores on the FS-ADS and ZBI relative to spousal caregivers, as mentioned previously, the gender distribution of our sample makes it difficult to interpret this difference.

The reasons that stigma and burden are greater in female caregivers are unclear. One possibility is that the physical tasks of caregiving, such as lifting or dressing, are more burdensome for women. It is also possible that female caregivers may have more homemaking responsibilities and that the addition of a caregiving role further adds to the burden of these responsibilities. If so, feelings of burden in men versus women may evolve as male and female roles become less traditional in future generations. It is also possible that men are simply less likely to report feelings of stigma or burden.

One study [14] has described gender differences in the approach to caregiving. This study suggests that caregiving husbands use a task-oriented approach, while caregiving wives are more emotionally focused. In one study, six strategies that caregiving husbands employed were identified: exerting force, focusing on tasks, blocking emotions, minimizing disruption, distracting attention, and self-medicating [14]. It appears that these gender differences may have implications on caregiver feelings of burden and stigma.

Female spousal caregivers may also suffer more stigma, as well as burden, because studies have suggested that caring for men with dementia is more burdensome. Men with dementia tend to have more behavioral symptoms, such as disinhibition, aggression, and sexual inappropriateness, than women with dementia [15]. These behaviors may be particularly stressful or embarrassing for caregivers and increase their feelings of stigma and therefore burden as well.

The present study would suggest that providing support for caregivers is crucial, particularly for female and adult child caregivers who appear to experience higher levels of stigma and burden. The current correlational data do not permit causal conclusions but do indicate a relationship between stigma and burden that bears further exploration.

### 5.1. Limitations

The skewed gender distribution across caregiver types in our sample makes it difficult to evaluate the effect of caregiver type on stigma and burden scores and also precludes an assessment for a caregiver type by gender interaction. Future work examining these relationships is warranted.

There was a small but statistically significant difference between mail and telephone responders for FS-ADS-C score (*t*(80) = −2.04, *p* = .047), but not ZBI score (*t*(80) = −0.32, *p* = .749), suggesting that response method may have had some influence on participants' pattern of responding. However, our primary finding of female gender being associated with higher reported levels of caregiver stigma remained when testing mail and telephone response groups separately, as did the positive correlation between caregiver stigma and caregiver burden (all *p* < .05; data not shown). Females reported greater caregiver burden relative to males in both phone response and mail response groups as well, although this result did not maintain statistical significance in the latter group.

Finally, the FS-ADS had to be modified, as described in Section 2.3, to accommodate spousal caregivers. As a result, we were unable to use the exact, validated measure.

### 5.2. Conclusions

Despite these limitations, our findings highlight the relationship between the stigma and burden associated with caring for a family member with a memory disorder. We have also demonstrated for the first time that female and adult child caregivers in particular are vulnerable to greater levels of both stigma and burden. Future research should also examine patient gender and its relationship with caregiver stigma and burden to further identify the characteristics of caregivers who are at risk for feelings of stigma and burden.

Given our study's results, strategies designed to reduce stigma at the personal, group, or societal level may also help reduce caregiver burden, particularly for female and adult child caregivers. Caregivers in the study identified public education about dementia and increased communication with persons with dementia as potential strategies to reduce stigma. Future research should attempt to further develop interventions and test them for efficacy.

## Figures and Tables

**Table 1:** Caregiver characteristics by caregiver type.

	Spousal caregivers	Adult child caregivers	*p* value
	(*n* = 59)	(*n* = 23)	
Caregiver age, mean (SD)	76.6 (7.9)	56.3 (7.0)	<.001^a^
Caregiver gender, *n* (%)			
Male	21 (35.6)	3 (13.0)	.044^b^
Female	38 (64.4)	20 (87.0)	
Caregiver years of education, mean (SD)	16.0 (2.9)	16.2 (3.2)	.803^a^
Live with person with dementia, *n* (%)			
Yes	52 (88.1)	6 (26.1)	<.001^b^
No	7 (11.9)	17 (73.9)	
Diagnosis of patient, *n* (%)			
Alzheimer's	27 (45.8)	8 (34.8)	.143^c^
“Dementia”	26 (44.1)	12 (52.2)	
Not diagnosed, or other	5 (8.5)	3 (13.0)	
Missing	1 (1.7)	0 (0.0)	
Years since symptoms first noticed in patient, mean (SD)	5.9 (3.7)	5.3 (3.2)	.554^a^
Years since diagnosis in patient, mean (SD) (*n* = 76)	3.8 (2.6)	4.0 (2.2)	.744^a^

^a^ *p* value is based on independent *t*-test.

^b^ *p* value is based on Chi-square test.

^c^ *p* value is based on Fisher's exact test.

**Table 2:** Caregiver stigma and burden, by caregiver type and gender.

Measure	All caregivers (*n* = 82)	By caregiver type			By caregiver gender		
		Spousal caregivers (*n* = 59)	Adult child caregivers (*n* = 23)	*p* value^a^	Female caregivers (*n* = 58)	Male caregivers (*n* = 24)	*p* value^a^
FS-ADS-C (M (SD))	20.5 (4.9)	19.6 (4.2)	22.7 (6.0)	.034	21.7 (5.1)	17.7 (3.1)	<.001
ZBI (M (SD))	17.7 (9.3)	16.0 (8.7)	21.9 (9.8)	.010	19.4 (9.3)	13.5 (8.0)	.009

^a^ *p* value is based on independent *t*-test for continuous variables.

## References

[1] Alzheimer's Association (2015). 2015 Alzheimer's disease facts and figures. *Alzheimer's & Dementia*.

[2] Gitlin L. N., Schulz R., Prohaska R. T., Anderson L. A., Binstock R. H. (2012). Family caregiving of older adults. *Public Health for an Aging Society*.

[3] Carretero S., Garcés J., Ródenas F., Sanjosé V. (2009). The informal caregiver's burden of dependent people: theory and empirical review. *Archives of Gerontology and Geriatrics*.

[4] Etters L., Goodall D., Harrison B. E. (2008). Caregiver burden among dementia patient caregivers: a review of the literature. *Journal of the American Academy of Nurse Practitioners*.

[5] Goffman E. (1963). *Stigma: Notes on the Management of Spoiled Identity*.

[6] Werner P., Mittelman M. S., Goldstein D., Heinik J. (2012). Family stigma and caregiver burden in Alzheimer's disease. *Gerontologist*.

[7] Batsch N. S., Mittelman M. S., Alzheimer's Disease International (2012). *World Alzheimer Report 2012: Overcoming the Stigma of Dementia*.

[8] Liu D., Hinton L., Tran C., Hinton D., Barker J. C. (2008). Reexamining the relationships among dementia, stigma, and aging in immigrant Chinese and Vietnamese family caregivers. *Journal of Cross-Cultural Gerontology*.

[9] Werner P., Heinik J. (2008). Stigma by association and Alzheimer's disease. *Aging & Mental Health*.

[10] Werner P., Goldstein D., Buchbinder E. (2010). Subjective experience of family stigma as reported by children of Alzheimer's disease patients. *Qualitative Health Research*.

[11] Werner P., Goldstein D., Heinik J. (2011). Development and validity of the Family Stigma in Alzheimer's Disease Scale (FS-ADS). *Alzheimer Disease & Associated Disorders*.

[12] Bédard M., Molloy D. W., Squire L., Dubois S., Lever J. A., O'Donnell M. (2001). The Zarit Burden interview: a new short version and screening version. *The Gerontologist*.

[13] Chang K. H., Horrocks S. (2006). Lived experiences of family caregivers of mentally ill relatives. *Journal of Advanced Nursing*.

[14] Calasanti T., King N. (2007). Taking ‘women's work’ ‘like a man’: husbands' experiences of care work. *The Gerontologist*.

[15] Brodaty H., Connors M. H., Xu J., Woodward M., Ames D. (2015). The course of neuropsychiatric symptoms in dementia: a 3-year longitudinal study. *Journal of the American Medical Directors Association*.

